# A Century of Medical History in the County of Erie.—1800–1900

**Published:** 1899-04

**Authors:** William Warren Potter

**Affiliations:** Buffalo, N. Y.


### A CENTURY OF MEDICAL HISTORY IN THE COUNTY OF ERIE.—1800–1900.

By WILLIAM WARREN POTTER, M. D., Buffalo, N. Y.

*Pioneer Physicians—Medical Societies—Medical Colleges—Hospitals—Medical Journals—Women Physicians—History of Homeopathy—Medical Officers of the Civil War—Individual Members of the Profession.*

[*Continued from the March edition.*]

IN THE foregoing pages it has been the aim to give a statement of facts with reference to the history of medicine in Buffalo and Erie county during the past 100 years. It has also been the purpose to deal principally with first things giving in detail the organisation of medical societies, hospitals, institutions and everything connected in a public manner with medicine. At the outset it was also necessary to write somewhat elaborately of individual members of the medical profession, since they alone in the early days constituted its guild.

As a conclusion, however, it seems proper to deal with medicine in a broader aggregate, showing what has been accomplished during the century now drawing to its close and in which the physicians of Erie County played an important part. Jenner had but just discovered vaccination when the history of medicine in Erie county began, and the physicians of that early day, at first slow to adopt it as a preventive or amelioration of smallpox, were among the earliest champions of this discovery and some of them lived to see it put into general practical application as well as to witness the realisation of this great triumph of preventive medicine.

Singularly enough, after Jenner's discovery it was almost fifty years, *i.e.*, nct until 1846, before the next great advance was made in the field of prevention. The discovery and application of anesthesia to the prevention of pain in surgical injuries and operations produced a more profound impression upon the medical world, if possible, than did vaccination. The first successful employment of anesthesia in surgery occurred in the Massachusetts General Hospital, at Boston, October 16, 1846, the same year that the Buffalo Medical College opened its doors for instruction. The teacher of surgery, Professor Frank Hastings Hamilton, began early to make use of anesthetics in his surgical work, and he played an important part in early establishing their use upon a practical basis. It was not long before Professor James P. White, Dr. George N. Burwell and others, acting on the suggestion of Sir James Y. Simpson, of Edinburgh, demonstrated the practical employment of anesthesia in the practice of obstetrics, which has led to an amelioration of the pangs of maternity. In Buffalo, too, demonstrative midwifery was first practised in America, a system that provoked criticism at first, but that since has been adopted everywhere as the only proper method of teaching that art. The courage displayed by Dr. White on the historic occasion referred to has served to give his name distinguished prominence in connection with the teaching of midwifery, and to make Buffalo famous as the place of its origin.

The exact method adopted by Professor Frank Hastings Hamilton in the treatment of fractures and the perfection of measurements which he insisted upon, served to reduce resulting shortening and deformities to a minimum. During the fifteen years of his residence in Buffalo and while he was teacher of surgery at Buffalo University Medical College he laid the foundation for perfected methods in the management of bone injuries. He also wrote a treatise on fractures and dislocations that became a text-book in nearly all the medical colleges in this country and in Europe and which has been translated into several foreign tongues. He was a disciple of Sir Astley Cooper and of Masoneuve, a forceful and accomplished teacher, a scholarly man and a distinguished surgeon. He left the imprint of his teaching on the medical profession of Buffalo in a lasting manner.

The clinical study of diseases of the chest was first reduced to precision by Dr. Austin Flint while he was a resident of Buffalo and a teacher in the medical college. He had a musical ear, capable of detecting abnormal chest sounds in the minutest degree, and he was the originator of methods in the teaching and practice of internal medicine that attracted the attention of the professional world. Dr. Flint also discovered the fact that typhoid fever was a water-borne disease and startled the world by his announcement of that fact. He became one of the most distinguished physicians of his time and left a lasting impression on the annals of medicine.

It was in Buffalo that the teaching of physiology was first in this country reduced to an exact science through vivisection and laboratory experiments under the masterly hand of Professor John C. Dalton. He came to Buffalo fresh from the pupilage of M. Claude Bernard, of Paris, whose methods he adopted. He attracted the attention of students of physiology everywhere and was soon invited to New York, where he continued his great work in an enlarged field. Physiology at once took rank as a foremost science which will always bear the impress of the name of Dalton.

The establishment of a medical college in Buffalo in 1846 has contributed largely to the professional development of Western New York and especially of Buffalo and Erie county. By far the larger number of physicians of this region are its graduates and it still flourishes in its new home with enlarged facilities for teaching, as one of the most respected medical colleges in the land. Its younger sister, Niagara University Medical College, established in 1883, took from the start a high ground with reference to medical education and increased length of college terms. It succeeded in making its principles recognised and patterned after. These two schools were a credit to the medical profession and the age. And when they were amalgamated in June, 1898, they further accentuated the principles that had governed both from their inception. The faculties of these two schools acting together in a common cause must of necessity make a stronger college than when working separately with divided interests.

The medical societies in Buffalo and Erie county have also exerted a beneficial influence in training physicians to thought and action and in making them abler and better exponents of established medicine. We have in these pages devoted considerable space to the several medical societies because, after all, these are the important avenues through which medicine marches to its appointed place in history. When a man becomes a frequent contributor of papers to his local medical society and is ready to take part in the debates that arise from such contributions, he establishes himself in the minds and hearts of his fellow-citizens as a progressive physician entitled to confidence. Such papers and debates when printed, as they should be, in the home medical journal at once become a part of the history of the medical profession of that region.

During the century just closing, medicine has advanced so markedly that it has hardly been possible to keep pace with its strides. But it is a gratifying fact to be able to record in these pages that the guild in Buffalo and Erie county has kept the faith and stands abreast in intelligence and science with its professional brethren in any quarter of the globe.

An enumeration of some of the more prominent inventions and discoveries made during the closing century relating to the medical profession and which have contributed to the cure of disease and the lengthening of human life will, in this connection, not be uninteresting. Without attempt at chronological accuracy the first to be mentioned is vaccination, then chloroform, next the stethoscope, which has so developed and perfected the study of the most obscure maladies of the chest; the endoscope, the laryngoscope, the otoscope, the ophthalmoscope and the speculum, all instruments used in lighting up portions of the interior of the body; the clinical thermometer, which guides almost unerringly in discerning the nature and severity of many diseases; the sphygmograph, that makes the pulse write out the story of the heart throbs; the marvelous revelations of the microscope; the hypodermic syringe, which enables us to treat many diseases inaccessible to therapeusis through the ordinary channels; the aspirator, which aids in the diagnosis and treatment of many surgical diseases; the Esmarch bandage, that permits the most important operations without the loss of blood; the development and perfection of operations in the various cavities of the body—cranial, thoracic, abdominal and pelvic—for diseased conditions, new growths and injuries; the discovery and application of the principles of asepsis and antisepsis as related to surgical and obstetrical procedures; the study of the germ theory of disease in connection with the science of bacteriology; increased knowledge and improved methods in public health administration and general sanitation relating to the prevention of disease; the application of the principles of higher medical education as related to methods of teaching and lengthened terms of study; the separate examination by the state, apart from and independent of the colleges, for license to practise medicine; and finally the x-ray and other electrical devices that are useful in the study and treatment of surgical diseases. These, together with a refined chemistry and perfected pharmacy, are among the contributions of the XIXth Century to the glories of medicine.

It is gratifying to have lived in such a period and to be able to record the fact that the medical profession of Buffalo and Erie county has not only witnessed these advances but has taken an active part in either discovering, developing or perfecting many of them.

[*Concluded next month.*]

